# Neural bases of ingroup altruistic motivation in soccer fans

**DOI:** 10.1038/s41598-017-15385-7

**Published:** 2017-11-23

**Authors:** Tiago Bortolini, Patrícia Bado, Sebastian Hoefle, Annerose Engel, Roland Zahn, Ricardo de Oliveira Souza, Jean-Claude Dreher, Jorge Moll

**Affiliations:** 1 Cognitive and Behavioral Neuroscience Unit, D’Or Institute for Research and Education, Rio de Janeiro, Brazil; 2 Graduate Program in Morphological Sciences, Federal University of Rio de Janeiro, Rio de Janeiro, Brazil; 3 Clinic for Cognitive Neurology, University of Leipzig, Leipzig, Germany; 4 Max Planck Institute for Human Cognitive and Brain Sciences, Leipzig, Germany; 5 Institute of Psychiatry, Psychology & Neuroscience, Department of Psychological Medicine, Centre for Affective Disorders, King’s College London, London, SE5 8AZ UK; 6 Neuroeconomics, Reward and Decision-making Team, Institut des Sciences Cognitives Marc Jeannerod, Centre National de la Recherche Scientifique, 69675 Bron, France

## Abstract

Humans have a strong need to belong to social groups and a natural inclination to benefit ingroup members. Although the psychological mechanisms behind human prosociality have extensively been studied, the specific neural systems bridging group belongingness and altruistic motivation remain to be identified. Here, we used soccer fandom as an ecological framing of group membership to investigate the neural mechanisms underlying ingroup altruistic behaviour in male fans using event-related functional magnetic resonance. We designed an effort measure based on handgrip strength to assess the motivation to earn money (i) for oneself, (ii) for anonymous ingroup fans, or (iii) for a neutral group of anonymous non-fans. While overlapping valuation signals in the medial orbitofrontal cortex (mOFC) were observed for the three conditions, the subgenual cingulate cortex (SCC) exhibited increased functional connectivity with the mOFC as well as stronger hemodynamic responses for ingroup versus outgroup decisions. These findings indicate a key role for the SCC, a region previously implicated in altruistic decisions and group affiliation, in dovetailing altruistic motivations with neural valuation systems in real-life ingroup behaviour.

## Introduction

The capacity to develop strong social bonds to genetically unrelated group members may have played a central role in hominin evolution and in gene-culture coevolution^1,2^. Group belongingness is considered a basic human need^3^ and behavioural research has demonstrated the human tendency to favour ingroup over outgroup members, even when groups are defined by arbitrary surface features like those created in the laboratory^4,5^. Such important advances on the understanding of the psychological mechanisms of ingroup bias and ingroup-outgroup categorization^5,6^ have recently been bolstered by neurobiological studies^7,8^. These lines of evidence, however, fall short of explaining the unique motivational capacity of humans to incur personal sacrifices to benefit genetically unrelated members of culturally defined groups.

Previous fMRI studies addressing the neurobiology of ingroup altruism have mainly employed arbitrary groups, based, for example, on a bogus perceptual task or personality test forming groups indicated by different colors^9,10^. Recent studies, however, have investigated natural social identities built on long-lasting, strong ties based on daily engagement^6^ (such as ethnical groups^11^ and university affiliation^12^), inferring participant’s altruistic motivations from their behaviour in classic economic games. These studies showed increased responses in the ventral striatum (VS), a key region of the reward system^13^, for ingroup donations compared to self-rewards^11^. In addition, higher self-reported scores on the motivational components of a group identification scale positively correlated with VS responses when comparing ingroup vs. outgroup gains^12^. The VS is also activated by decisions to donate anonymously to charitable organizations^14,15^ and by stimuli representing attachment figures such as kin and romantic partners^16,17^. Another core component of the reward system is the medial orbitofrontal cortex (mOFC)^13^. The mOFC is engaged by the subjective value of choice alternatives^18,19^, during anticipation of rewards and at the time of rewarded outcomes^20–23^. This region is also intimately involved in social cognition^24,25^, and during in group categorization and pro-group behaviour^8,26,27^.

In addition to the evidence pointing to the reward system in ingroup cooperation and social valuation^28–30^, the subgenual (or subcallosal) cingulate cortex (SCC; BA25 and parts of BA24/32 and 33) has been more specifically implicated in altruistic decisions^14,31–33^ and prosocial learning^34^. Interestingly, the SCC was also engaged during evaluation of ingroup-related stimuli in contexts that did not involve decisions^35–38^. The SCC receives direct hypothalamic projections from oxytocin and vasopressin-releasing neurons and is populated with OT receptors^39–42^. These neuropeptides and their direct action in the basal forebrain are crucial for social bonding and other attachment-related behaviors^43,44^. Taken together, these lines of evidence suggest that the SCC may be an important neural component for enabling culturally-defined ingroup attachment and behaviour^37,43–45^.

Attachment to cultural groups is a human universal that is powerfully manifested in social mass phenomena, such as in soccer fandom, even in the absence of kinship and moral values. Soccer fans fulfil the criteria for a “natural group”^46^, showing strong group attachment in real-life settings that often translate into costly behaviours—from buying expensive tickets and going to away matches to engaging in street brawls^47–50^. Soccer rooting therefore provides a unique instance of culturally defined ingroup belongingness. Yet, the neural underpinnings of altruistic motivation toward culturally defined groups in more naturalistic and meaningful settings remain to be further characterized.

**Figure 1:**
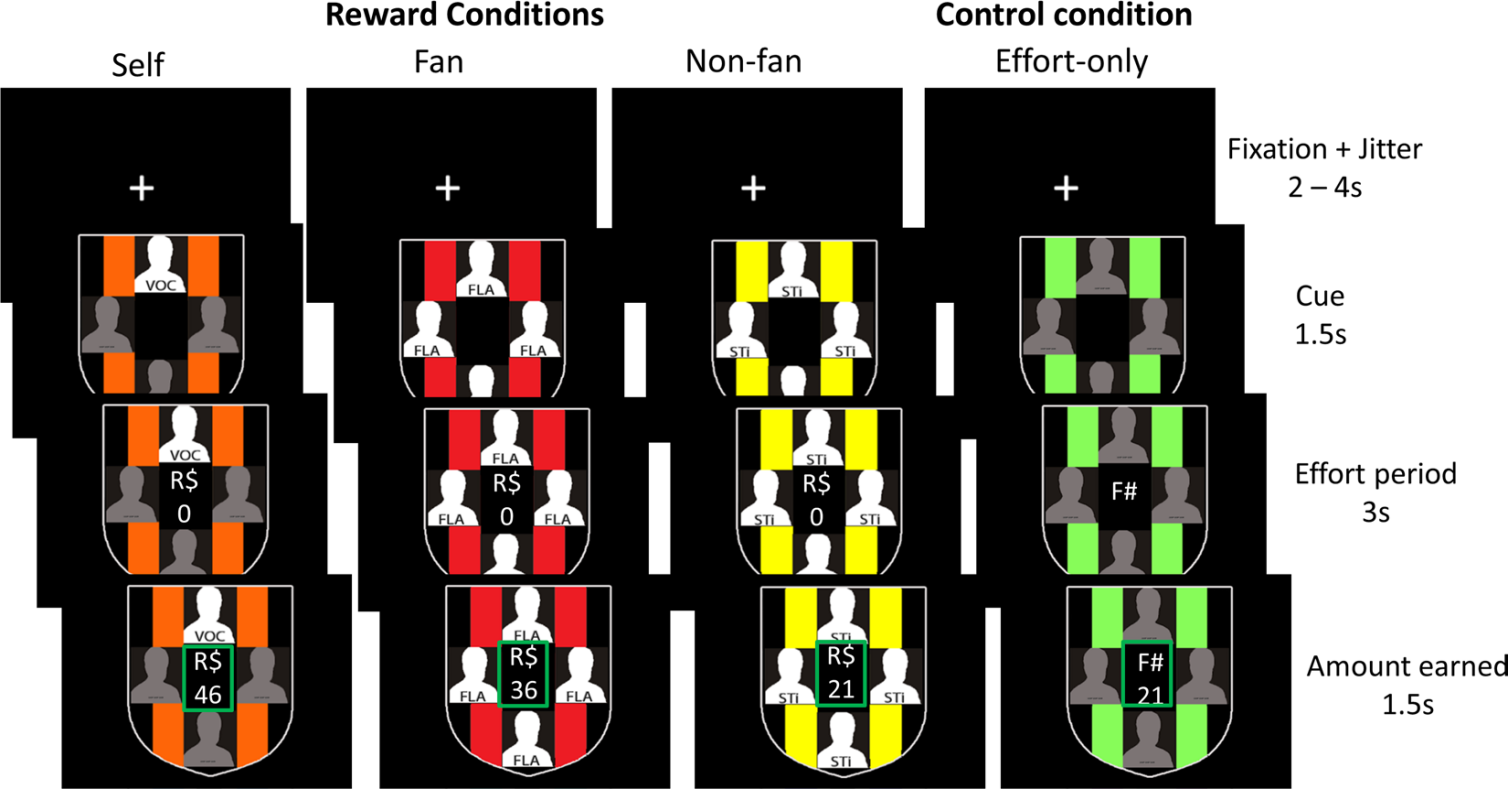
fMRI task design. A cue indicating trial type was presented after a fixation cross jitter period. After cue presentation, participants pressed the handgrip dynamometer (effort period for Reward and Effort-only conditions) or passively observed the cue (in Cue-only conditions, not shown here, but see Fig. S1 for detailed description of the fMRI task). Real-time feedback of the amount earned during the effort period was provided, followed by a green outline indicating the end of the trial and the amount earned. The first three columns depict the Reward conditions (Self, Fans and Non-fans). The fourth column depicts an example of an Effort-only control condition. Each Reward condition was matched to its specific Effort-only control condition. The cue for the Effort-only condition was always the same, and indicated that participants should reach the same effort as in the preceding Reward trial (as indicated by the number at the centre), but in the absence of monetary incentive. The letters on the silhouettes stands for: VOC, “*você”* (Self); FLA, “*Flamengo”* (one of the soccer teams); STI, “*Sem Time”* (Non-fans).

Here, we frame soccer fandom as a naturalistic model of group membership to investigate the neural mechanisms underlying ingroup attachment and altruistic motivation, which entails the ultimate goal of increasing a third party’s welfare^51^. To this aim, we carried out an event-related fMRI experiment on highly identified male soccer fans (N = 27) with a novel behavioural measure tapping on the motivation to earn money in three main reward conditions: (i) earning for oneself (self-reward, or “Self”, condition); (ii) earning for other fans of their own soccer team (“Fans” condition); and (iii) earning for “neutral” participants, that is, participants who are not supporters of soccer clubs (“Non-fans” condition). The monetary rewards were associated with the amount of physical effort participants applied to an electronic handgrip dynamometer (Fig. 1; see also Fig. S1 for a detailed depiction of the fMRI task). Considering motivation as the “*process which facilitates overcoming the cost of an effortful action to achieve the desired outcome*”^52^, this paradigm provided a measure of participants’ actual motivation at the time of the decision to spend physical effort for earning money in all experimental conditions (rather than simply requiring psychological decisions that resulted in a future monetary outcome). The Self, Fans, and Non-fans conditions, respectively, enabled us to assess motivations associated with self-gains, ingroup altruism and outgroup altruism^53^ by way of physical work. Additional conditions (detailed below) provided tight controls for physical effort, task cues and associated reward expectancy.

We hypothesized that motivation to obtain rewards for oneself and for fellow fans would engage brain regions associated with valuation (especially the medial orbitofrontal cortex [mOFC] and the ventral striatum [VS]) more than rewards for non-fans^13,20,54,55^. We expected that motivation to make an effort to benefit fellow fans compared to non-fans (while controlling for the effects of effort *per se*) would elicit increased coupling between the mOFC, a key region to valuation processes^20,23,55^, and the SCC, a region that has been specifically implicated in altruistic^28,35–38^ and affiliative behaviours^16,35,37,38,56^. We additionally predicted that the SCC would be more strongly engaged by altruistic motivation to benefit ingroup fans compared to non-fans.

## Results

### Behavioural results

**Figure 2:**
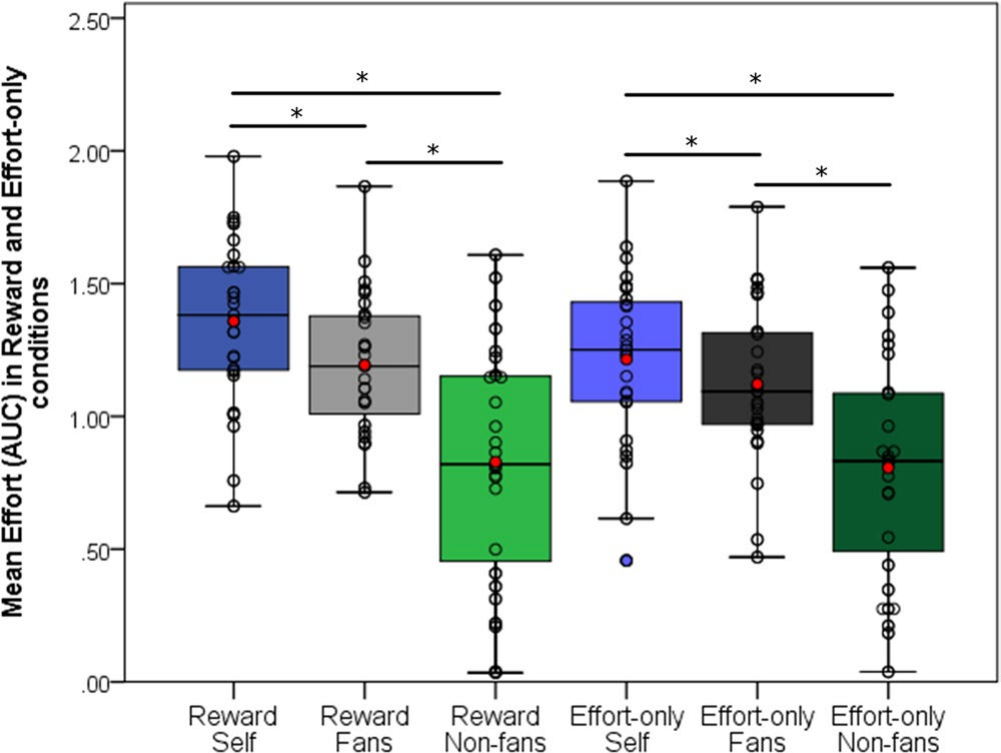
Behavioural results. Box-plots and data points (N = 28) of mean effort (AUC: area under the curve) applied in each task (Reward and Effort-only) and the three conditions (calibrated according to each participant’s maximum grip force) showing that there were significant differences in effort between all three Reward conditions (*p < 0.001). Red dots represent the mean in each condition.

A two-way repeated measures ANOVA was conducted to compare the main effects of Monetary Incentive (Reward, Effort-only) and Group (Self, Fans, Non-fans) and its interaction on the effort applied to the handgrip. The interaction effect between Monetary Incentive and Group was significant [*F*(2,52) = 6.98, *p* = 0.002, partial η² = 0.21)]. Analysis of 95% confidence intervals showed that there were no overall statistical differences between Reward and Effort-only conditions, indicating that the amount of effort employed in the Effort-only conditions did not statistically differ from their respective Reward conditions (Fig. 2). This is consistent with our instruction requiring participants to put the same effort on Effort-only trials as they did in the Reward trials. In contrast, paired samples *t*-tests found significant differences between all Reward conditions (α set at 0.016, two-tailed, Bonferroni adjustment for family-wise error [FWE]). Participants made more effort to obtain money for themselves (M = 1.36, 95% CI [1.23, 1.48]) compared to Fans (M = 1.19, 95% CI [1.08, 1.30], *t*(26) = 3.38, *p* = 0.002) and Non-fans (M = 0.86, 95% CI [0.65, 1.01], *t*(26) = 6.82, *p* < 0.001), and invested more effort to benefit fans than non-fans (*t*(26), *p* < 0.001) (Fig. 2). Spearman Rho correlations indicated no significant correlations between group alignment self-report mean scores and mean effort on the task (all p’s > 0.09).

### Common effects of Self-concerned and Altruistic Reward conditions

**Figure 3:**
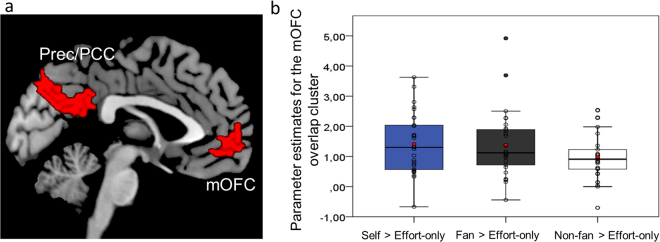
Common effects of Self-concerned and Altruistic Reward conditions. (**a**) Conjunction analysis of common effects, i.e. graphical overlap of thresholded statistical maps (voxel level, *p* < 0.001; cluster-corrected FWE, *p* < 0.05 at the whole brain level) of Reward versus Effort-only conditions showing clusters encompassing the medial orbitofrontal cortex (mOFC) and the precuneus/posterior cingulate cortex (Prec/PCC, which was not *a priori*-defined). (**b**) Box-plots and data points (N = 27) of parameter estimates for each Reward > Effort-only contrast of the medial orbitofrontal cortex (mOFC) cluster. Red dots represent the mean parameter estimates in each contrast.

One sample *t*-tests were performed for each Reward condition compared to their respective Effort-only condition (Tables S1–S3 and Figs. S2–S4). To check the overlap between the clusters that survived FWE cluster correction among these contrasts, the thresholded *t*-maps were subjected to a conjunction analysis (logical AND between thresholded maps^57^) (see Methods). All three contrasts displayed common brain activation in the mOFC, the precuneus and the posterior cingulate cortex (PCC) (Fig. 3a). A repeated measures ANOVA of the parameter estimates extracted from the overlapped mOFC cluster of each Reward > Effort-only contrast indicated no pairwise statistical difference between the contrasts (Fig. 3 b).

### Functional connectivity analysis (FC)

**Figure 4:**
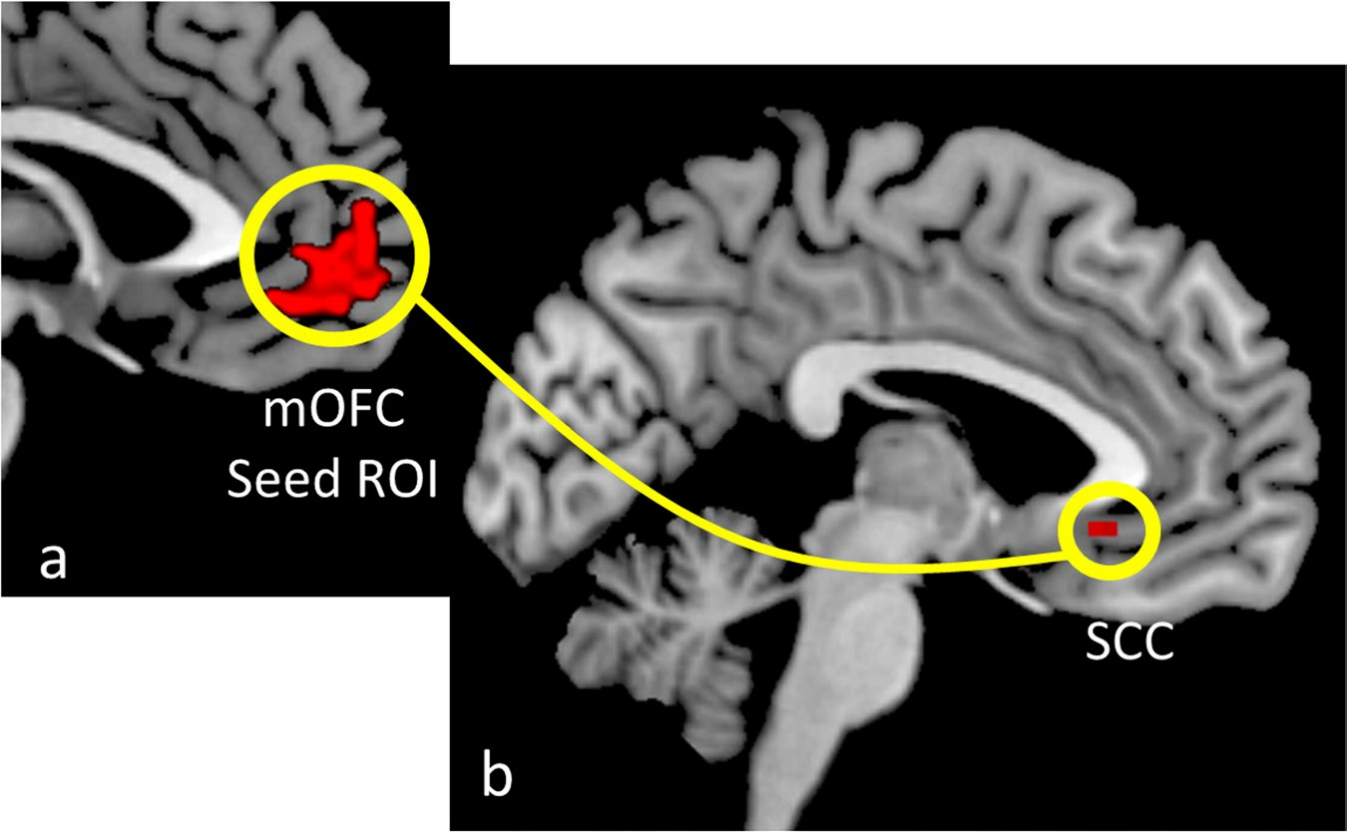
Connectivity between mOFC and SCC. (**a**) Medial orbitofrontal cortex (mOFC) cluster shown in Fig. 3a was employed as seed ROI. (**b**) Paired *t*-test of Fans > Non-fans *Z*-transformed correlation maps from seed ROI showing increased functional connectivity between the mOFC and the subgenual cingulate cortex (SCC). Whole-brain image in (**b**) is displayed at an uncorrected threshold of *p* = 0.005 (k = 5 voxels) for visualization purposes.

Based on the role of the mOFC in valuation and decision making^23,55^, we investigated whether mOFC valuation signals were specifically coupled with SCC activity during ingroup altruistic behaviour. A seed-to-voxel FC analysis using beta-series correlations (BSC) was employed for this purpose. BSC FC is considered to be more appropriate for event-related designs than conventional psychophysiological interaction methods^58^ (see Methods). The seed mOFC ROI was based on the overlap among the three Reward > Effort-only contrasts (Fig. 4 a). The contrast Fans > Non-fans showed higher connectivity of mOFC with our *a priori* SCC ROI (*p* < 0.05, FWE-corrected SVC; *Z* = 3.41; local maxima [0; 26; −7]; Fig. 4 a). A control mOFC-SCC FC analysis comparing Effort-only Fans > Effort-only Non-Fans showed no significant differences, indicating that the increased connectivity in the Fans > Non-Fans contrast could not be attributed to different levels of effort.

### Differential effects for Self and Altruistic Reward conditions

**Figure 5:**
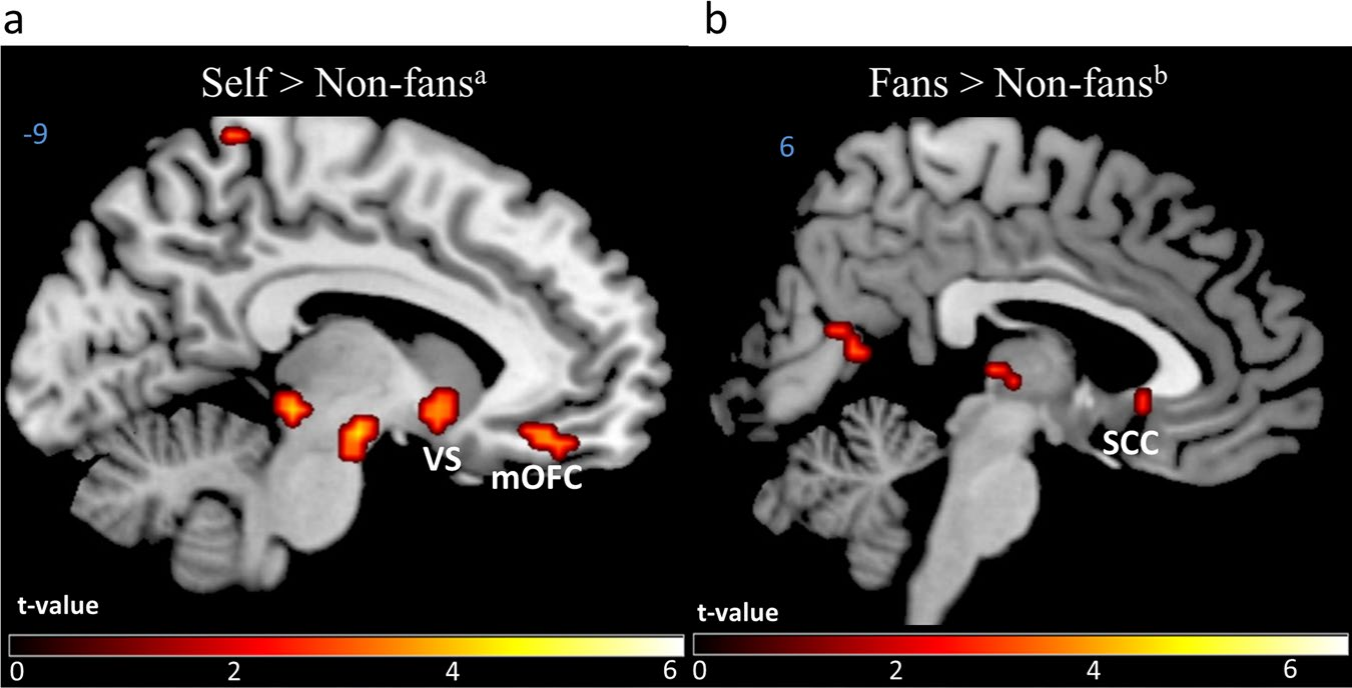
Differential brain responses for each Reward conditions controlling for the Effort-only conditions. (**a**) Increased BOLD signal in the right and left ventral striatum (VS) and the medial orbitofrontal cortex (mOFC; SVC FWE-corrected, p < 0.05, cluster level) for Self vs. Non-fans contrast (controlling for Effort-only). (**b**) Increased BOLD signal in subgenual cingulate cortex (SCC) spreading to the caudate nucleus (SVC FWE-corrected, p < 0.05) for Fans vs. Non-fans contrast (controlling for Effort-only). Statistical maps are displayed at uncorrected *p* = 0.005, k = 5 voxels, for visualization purposes. ^a^[Self − Effort-only] – [Non-fans − Effort-only]; ^b^[Fans − Effort-only] − [Non-fans − Effort-only].

To search for specific brain responses to each Reward condition controlling for the effort *per se* paired *t*-tests using the first level contrasts images of each Reward versus Effort-only conditions were performed (e.g. [Fans – Effort-only] – [Non-fans – Effort-only]; see Methods). In line with our *a priori* hypotheses, analysis using small volume correction (SVC) over *a priori* ROIs^14,35,37^ revealed that making effort for Self vs. effort for Non-fans led to increased responses in the VS and mOFC, but not in the SCC (Table 1 and Fig. 5 a). Contrasting Fans to Non-fans showed higher activation in the SCC (Table 1 and Fig. 5 b), but not in the VS or mOFC. However, the SCC cluster did not survive Bonferroni alpha level adjustment (*p* < 0.016) for the number of ROIs tested. As expected, the remaining contrasts (Self > Fans, Fans > Self, Non-fans > Fans and Non-fans > Self) revealed no significant activations in *a priori* ROIs at the reported statistical levels. Whole-brain results (*p* < 0.001 uncorrected voxel-level, cluster FWE corrected) are also reported in Table 1. A supporting analysis controlling for the effects of the cues was conducted to exclude the possibility that cue type could have influenced the main results reported above. No cluster survived in any *a priori* ROIs even at a lenient threshold of *p* > 0.05 (uncorrected; voxel level) when contrasting Cue-only conditions against each other.

**Table 1:** Whole-brain and small volume correction clusters of differential effects of each Reward condition controlling for the effort.

Region	*k*	*Z*	*p*_c_	x	y	z
**(Self - Effort-only) > (Non-fan – Effort-only)**						
Precentral Gyrus*	87	5.60	<0.01	−45	−13	41
Left Accumbens^†^	46	3.63	<0.01	−12	11	−7
Medial orbitofrontal cortex^§^	11	2.88	0.04	−9	38	−10
**(Self - Effort-only) > (Fan - Effort-only)**						
Lateral Occipital Cortex inferior division^*^	44	4.90	<0.01	48	−73	−1
**(Fan - Effort-only) > (Self - Effort-only)**						
Cuneal Cortex^*^	127	5.50	<0.01	0	−85	26
Lingual Gyrus^*^	127	4.40	<0.01	0	−88	−4
**(Fan - Effort-only) > (Non-fan - Effort-only)**						
Insular Cortex^*^	68	6.05	<0.01	36	−4	8
Subgenual cingulate cortex^§^	9	3.02	0.05	6	23	2

*Note*. Clusters survived either whole-brain (*p* < 0.001 uncorrected voxel-level, cluster FWE corrected) (*) or small volume correction over an *a priori* anatomical ROI, corrected for the number of ROIs (^†^). Regions surviving small volume correction over anatomical ROIs but which did not survive Bonferroni correction for the three *a priori* ROIs are also listed (^§^). *A priori* ROIs coordinates: SCC [0, 26, −5]^14,101^; mOFC [−2, 40, −4]^27^; and VS [12/−12, 10, −6]^28^. *k* = cluster extent; *p*_*c*_ = p-value cluster level FWE; *x*, *y*, *z* = MNI coordinates.

## Discussion

Here we examined fMRI responses of soccer fans during self-concerned, ingroup altruistic and outgroup altruistic decisions in the context of soccer team belongingness. We employed handgrip effort to tap on altruistic motivations^52^ to benefit ingroup and a neutral group in a non-competitive altruistic context. Our design also included an Effort-only condition not associated with monetary rewards. Some previous fMRI studies that specifically investigated ingroup altruism have employed arbitrary groups^9,10^. Such groups, however, have low social meaning. Such meaning is a central ingredient of group belongingness typical of real-life social contexts^59^, and is robustly present in soccer fandom across the world, especially in Brazil. Apart from anecdotal cases, such as Brazilian fans selling their own furniture and house appliances to attend an away match in another continent^60^, another striking example of the relevance and the meaning of soccer fandom to highly identified fans is the increased heart attacks reported in locations hosting important games^61–64^. In addition to the research focusing on minimal groups, studies focusing on natural groups^11,12^ have used classical economic paradigms, which lack a direct measure of prosocial motivation at the moment of the decision^65–68^. The study by Hackel *et al*. (2017), for instance, focused mainly on a passive task in which students witnessed monetary gains of two confederates who they believed were ingroups or outgroups.

Thus, previous studies have not provided direct evidence for the neural basis of altruistic motivation toward culturally defined, highly meaningful, anonymous ingroup members. Together, our main results demonstrated that (1) the mOFC, a key hub for valuation mechanisms, shows responses to motivation contingent on both self-concerned and other (vicarious) reward outcomes; (2) the mOFC had a stronger functional connectivity with the SCC in the Fans as compared to the Non-fans condition; (3) the SCC was more responsive in the Fans vs. Non-fans condition, whereas the VS and mOFC were more engaged by Self vs. Non-fans; and (4) these responses were not contingent on cue-related effects or on effort *per se*.

The mOFC was recruited in all Reward vs. Effort-only contrasts. This common effect across self-concerned and altruistic Reward conditions corroborates the evidence on the key role of this region in social cognition^24^ and in overall value computation, especially when costs are tied to outcomes^20–23^. Moreover, there was no statistical difference in the parameter estimates in the mOFC cluster among the three main Reward conditions, while controlling for Effort-only. This confirms a recent meta-analysis suggesting that mOFC responses are “person invariant”, non-selectively tracking different reward modalities (i.e., food, monetary, social) and the recipient of rewards (self or other)^69^. In addition to the mOFC, the overlap among all Reward conditions included another large cluster encompassing the precuneus and PCC. Although we have not predicted this effect, previous studies have implicated these midline parietal areas in ingroup bias^9,10^ and in other social cognition mechanisms of interpersonal psychological inferences^70^.

The SCC showed increased functional connectivity with the mOFC during ingroup trials in comparison to a neutral group, as shown by beta-series correlations analysis. As discussed before, the mOFC is closely implicated in valuation mechanisms, whereas the SCC has been implicated in group affiliation, prosocial emotions and altruistic decisions^14,20,23,32,69^. This increased mOFC-SCC functional connectivity was only observed when comparing Fans vs. Non-fans conditions. The fact that this effect could not be explained by physical effort *per se* and the lack of increased functional connectivity in the Self vs. Non-fans comparison indicates that the coupling of these regions may be specific for encoding altruistic motivation toward ingroups.

The SCC was most strongly engaged by ingroup altruistic effort compared to non-ingroup altruistic effort. However, this effect did not survive strict Bonferroni alpha adjustment over the three ROIs, and thus should be interpreted with caution. Our results expand previous findings by showing that the SCC is engaged in altruistic behaviour toward a highly meaningful group in comparison to a “neutral” group. It is worthy of note that although both conditions involved altruistic decisions leading to social rewards, they differed considerably in terms of group belongingness. Furthermore, effects in the left mid-insular cortex, which was not an *a priori* ROI, survived whole-brain analysis FWE cluster correction for the interaction contrast of Fans vs. Non-fans (see Fig. S6). This area has been consistently implicated in certain kinds of emotional experience, particularly in empathic distress^71–74^. The robust insular responses observed in our study suggest that it may also play a role in altruistic ingroup decisions. As expected, we found increased activity in the VS for the interaction contrast [Self – Effort-only] – [Non-fans – Effort-only], compatible with a hedonic response. Although previous studies have shown ingroup-related VS activation^11,12^, responses in the VS for the interaction [Fans – Effort-only] – [Non-fans – Effort-only] did not reach significance in our study (Fig. S5).

The increased mOFC-SCC functional connectivity and the differential activity of the SCC in association with prosocial behaviour directed to an ingroup compared to a neutral group provides new insights on the possible neural mechanisms underlying group belongingness. Considering the role of the mOFC for valuation of different outcomes and rewards^20,23,75^ and the evidence implicating the SCC in altruistic and affiliative behaviour^14,32,37,38^, it could be speculated that functional coupling between these regions may be important for integrating general valuation and hedonic mechanisms with affiliative behaviour in the context of meaningful social groups.

Although our focus was on Brazilian male soccer fans, we believe that our study has broader implications in the identification of the neural bases of ingroup altruistic motivation. Indeed, our effort manipulations to earn money allowed us to measure actual motivation. Moreover, the Self, Fans and Non-fans conditions enabled us to assess motivations associated with self-gains, ingroup altruism and outgroup altruism in a naturalistic situation (real affiliation with soccer team) *via* physical work.

Our task differs in important ways from those employed in previous studies involving intergroup and dyadic interactions. Decisions and associated physical effort were made to benefit a group of individuals or oneself, but not a specific ingroup or outgroup member. As mentioned before, recent studies addressing ingroup cooperation in natural groups focused either on the differences of prosocial decisions involving the self and specific other ingroup or outgroup members using a classic economic game^11,12^, or more specifically on empathy toward ingroups or outgroups^76,77^. Our design, however, focused on levels of prosociality and did not entail “ingroup-outgroup” conflict, as it would be the case if rival soccer clubs were employed. Although soccer also represents a good model for the study of parochial altruism—the human disposition toward costly ingroup prosocial behaviour and outgroup hostility^78–81^, our study focused on the altruistic motivations outside of competitive settings and outgroup hostility. It is also important to note that our paradigm did not rely on dyadic interactions (i.e., no faces or individual identities were used). Because our study was focused on group processes and in order to avoid excessive multiple comparisons, we have refrained from using measures that are typically employed in dyadic relations. Nonetheless, we cannot completely rule out the influence of individual traits relevant for altruistic motivation (such as empathy dimensions) on inter-subject variability^51^.

Our study employed an Effort-only condition to control for the effects of effort itself, allowing us to explore the motivational differences between the Reward conditions. This helps explain the lack of anterior cingulate cortex (ACC) responses, despite its consistent involvement in anticipated effort^82–86^.

We focused on highly identified fans to enhance affiliative, group-related responses. Consequently, there was little variation in psychometric measures of group belongingness, which is a possible explanation for the absence of statistical relationships between these scores and the effort measure, in addition to the low power due to the limited sample of the fMRI study. Further studies are needed to investigate the full spectrum of group belongingness, from loose to strong ties, its relationship to ingroup altruistic motivations and its neural basis. Moreover, considering the behavioural evidence indicating differences between males and females regarding ingroup behaviour, it will be interesting to explore whether these results would hold in a female sample^87–89^.

In summary, the SCC displayed increased connectivity with the mOFC in Fans vs. Non-fans condition indicating that even though overall valuation-related responses in the mOFC shared similarities among all three Reward conditions, the SCC was specifically engaged when altruistic motivations involved a highly relevant social group. The differential activity pattern of the mOFC, VS and SCC in Self, Fans and Non-fans conditions help illuminate the crossroads of the neurobiology of human altruistic motivation and ingroup belongingness.

## Methods

### Participants

The experiment was completed by 30 right-handed male volunteers (M_age_ 28.79 years, SEM = 1.41; M_education_ 15.20 years, SEM = 3.77) after providing written informed consent. They were recruited through social media and personal contacts. Participants should support one of the four most popular soccer teams in Rio de Janeiro (Botafogo, Flamengo, Fluminense or Vasco da Gama) and fit common inclusion criteria for fMRI research. All participants reported no history of psychiatric and/or neurological diseases, nor where taking any type of psychotropic medication. Data were acquired only with males for convenience reasons and because of unresolved gender differences regarding ingroup behaviour^87–89^.

One participant was excluded from fMRI analysis due to head movement (>3mm) and two others due to disbelief on the experimental paradigm (accessed afterwards during debriefing). Thus, the behavioural analysis included 28 participants (since one of the discarded participants was only due to movement in the MRI scanner) and the fMRI analysis 27 participants. The study was approved by the Research Ethics Committee board of the D’Or Institute (CEP 727.851) and all experiments were performed in accordance with relevant guidelines and regulations. A gift card on the average amount of money earned in the task described below was given to each participant (around R$40). None of the subjects have previously participated in an fMRI or economic experiment.

### Psychometric measures

In order to ensure that our fMRI sample represented fans with high team identification, a separate online pilot study (N = 401; M_age_ = 30.37; SD = 9.95; 52.4% females) was conducted to acquire normative scores for group alignment measures (see *SI text* for detailed description of each measure). Participants group belongingness was assessed with the following instruments: (1) the Brazilian version of the Football Supporter Team Identification Scale^90^ (e.g. “I strongly identify with the fans of my soccer team”); (2) an entitativity measure, which assess how much one perceives a given social group as an entity (e.g. “My soccer team fans have many characteristics in common”^91^); and (3) a psychological kinship measure^92^, which is the extent to which an individual perceives other group members as family (e.g. “Fans of my soccer team are like family to me”). Detailed descriptions of psychometric measures are provided in *SI text*.

### fMRI participants group alignment measures

Participants showing high identification with one of the most popular soccer teams in Rio de Janeiro, Brazil, were recruited for the fMRI study. These participants (N = 28; M_age_ = 28; SD = 6.89) scored higher in all alignment measures in comparison to the pilot study sample: *team identification* (M_pilot_ = 2.78 ± 1.50 vs. M_fMRI_ = 5.76 ± 0.95; *t*(429) = 10.71, *p* < 0.001), *psychological kinship* (M_pilot_ = 1.60 ± 1.01 vs. M_fMRI_ = 3.63 ± 1.54; *t*(429) = 10.17; *p* < 0.001), and *entitativity*, (M_pilot_ = 4.45 ± 1.64 vs. M_fMRI_ = 5.92 ± 1.47, *t*(429) = 4.76; *p* < 0.001). Participants reported that they felt “neutral” toward non-fans (M* = *3.55 ± 1.05, on a 7-point Likert scale ranging from 1 [“I see them as rivals”] to 7 [“I see them as fellow ingroups”], 4 being “Indifferent”) and they were less identified^93^ with non-fans (M* = *2.79 ± 1.69) than with fans (M* = *5.79 ± 1.11; *t*(28) = 8.55; *p* < 0.001). Participants filled in these measurers online, between 2 and 3 days before the scanning session, to ensure recruitment of highly identified fans only.

### Task and experimental design

In an event-related fMRI design, by pressing a dynamometer inside the scanner, participants could earn money for different individuals: themselves (Self condition), anonymous participants that supported the same soccer team (Fans condition) and anonymous participants that did not support any soccer team (Non-fans condition). Therefore, there were three main conditions varying the beneficiary of the effort put in the task. Importantly, the participants were instructed that the money earned would go to a pool of individuals (fans or non-fans) and after the completion of the experiment someone from the respective pool would be drawn to receive the mean amount earned *per* condition. Only in the Self condition participants were rewarded directly. The amount of effort made on each trial determined how much money was accumulated in each condition; these were called ‘Reward conditions’. Moreover, there were two control conditions: ‘Cue-only’ and ‘Effort-only’ conditions. In the former, participants only saw the cue representing each Reward condition, which were not followed by effort or monetary outcomes. In the latter, participants were asked to press the dynamometer as in the trial before, but without any monetary reward for self or others. In this case, participants were explicitly instructed that we were only interested in the effects of motor effort on brain activity. Therefore, these control conditions were treated as high-level baselines. Finally, to reinforce ingroup feelings, 15 movie clips of soccer fans of participants club were displayed pseudo-randomly interspersed across the experiment, as well as 15 movie clips of a ‘neutral’ group of fans in a similar context (i.e., a group of fans from a small team which the participants did not perceive as rivals). Because our experimental design focused on the altruistic motivation towards fellow fans as compared to a neutral group, the clips merely acted as an overall engagement strategy, and were therefore not further analysed. An LCD display mounted in the scanner room, which was seen by the participant by way of a mirror system adapted to the head coil, was used for stimulus delivery.

A dynamometer calibration phase before fMRI experiment was used in order to standardize the device for each participant maximum force. The calibration phase was presented as an ‘incentive task’ prior the experimental task, in which participants could earn an extra amount of money (R$50) on top of the mean accumulated in the experiment if they reached the number 50 presented in a screen. Based on pilot studies (N = 21), to win the extra money, participants would have to reach 40 kg in one of three trials, which was an extremely hard task. No participant earned the extra money and all reported that the incentive task had no influence on their performance and decisions on the experimental task. Participants’ maximum force was calculated as the mean force of the three trials during calibration. A more reliable approximation of participant’s actual maximum force was achieved by this calibration of maximum force involving a true reward.

Each trial of the experimental task consisted of a fixation cross presented for an average of 3000 ms (jittered; range = 2000–4000 ms with 250 ms increments), followed by a cue presented for 1500 ms indicating to which pool the money would be converted to or indicating an effort only condition (Fig. 1). For Reward conditions (Self, Fans and Non-fans), a ‘R$’ symbol was presented after the cue, followed by the number ‘0’ (zero); for the Effort-only conditions, ‘F#’ symbol was presented, followed by the number zero; for the Cue-only conditions, a ‘X%’ symbol was displayed, indicating that participants shouldn’t press the dynamometer and only watch the screen. In the Reward and Effort-only conditions, after the symbols were presented, 3000 ms were available to participants press the dynamometer as they wanted. Real-time feedback of how much participants were earning during each trial was displayed meanwhile pressure grip. The real-time feedback of the amount earned was proportional to the area under the curve of the trial, normalized by the participant’s maximum force obtained during calibration phase. For example, if a participant employed on average 80% of the maximum force during the effort period, he would earn R$40 out of the maximum amount (R$50) in that trial. The feedback value was updated every 100ms. After the effort phase, a green square around the final number indicating how much was accumulated in that trial was displayed for 1500ms. Notably, although previous studies used mainly binary choices as ‘decisions’, in our task ‘decisions’ were considered as the amount of effort one employed in each condition.

The experiment consisted of 120 trials: 72 trials per Reward condition (equally distributed across Self, Fans and Non-fans conditions), 24 trials for Effort-only (8 per Self/Fans/Non-fans) and 24 trials of Cue-only (8 per Self/Fans/Non-fans). Moreover, there were 10 baseline periods of 12 s (null events) and thirty video clips (range duration: 6 s–10 s), resulting in an overall total duration of ~38 min divided in three experimental runs of ~13 min. The preliminary amount of money accumulated in each main condition was displayed in the middle and after each run. After the scanning session, psychometric measures were filled again (there were no significant statistical differences in pre- and post-scan scores), and participants were then debriefed.

A detailed explanation-sheet describing the task was presented to participants before entering the scanner and they could ask any question to the experimenter. Additionally, a simulation of the task was performed in a computer screen to familiarize participants with the trial structure and the general procedure. Finally, after the calibration phase, there were 5 practicing trials inside the scanner for familiarization with the fMRI environment and further clarifications, if there were any remaining doubts.

Behavioural data analysis. The behavioural effects of condition on mean effort (mean AUC) were tested using repeated measures GLM in SPSS 22 (SPSS, Inc.). Šídák correction adjusted for multiple comparisons *post hoc* analyses of mean estimates were used to compare mean effort among conditions. Spearman Rho was used to evaluate possible relationships of psychometric measures and effort put in the task. A significance threshold (α) of 0.05, two-tailed, was adopted for all statistical tests, except when Bonferroni alpha adjustment for multiple comparison was applied.

### fMRI data acquisition and analysis

MRI data were acquired on a 3 T Achieva scanner (Philips Medical Systems) using a T2*-weighted echoplanar (BOLD contrast) sequence (TR = 2000 ms, TE = 22 ms, matrix = 80 × 80, FOV = 240 mm, flip angle = 90°, voxel size 3 × 3 mm; slice thickness = 3 mm, 40 slices, no gap; 256 volumes per run, 3 runs). Total functional scanning time was ~38 min. Before each run, five dummy volumes were collected for T1 equilibration purposes. A SENSE factor of 1.5 and dynamic stabilization were additionally used. These parameters were based on careful sequence optimization to maximize temporal signal-to-noise^94^ in brain regions that normally suffer from magnetic susceptibility effects, including the basal forebrain areas and ventromedial regions of the prefrontal cortex. High-resolution anatomical images were acquired with a 3D turbo field echo T1-weighted sequence (TR = 13 s, TE = 1.4 s, matrix = 256 × 356, FOV = 240 mm, slice thickness = 1 mm, 140 slices). Head motion was restricted by using foam padding and straps over the forehead and under the chin. Finally, respiratory cycle was recorded during fMRI acquisitions in order to avoid possible non-neural physiological changes as a result of the effort task.

The images were analysed using Statistical Parametric Mapping 12 software (SPM12; Wellcome Department of Cognitive Neurology, London, United Kingdom; www.fil.ion. ucl.ac.uk/spm). Motion correction was performed by realignment to the first image. All functional datasets underwent realignment, slice-time correction and normalization to the standard MNI space based on the coregistered anatomical segmentation. The original isotropic 3mm voxel resolution was kept constant. Functional data were smoothed using a 6 mm FWHM Gaussian spatial kernel.

Statistical analysis was performed using the two-stage mixed-effects GLM approach implemented in SPM12. At the first level analysis, box-car functions at stimulus onset for the different event types were convolved with SPM12’s hemodynamic response function to form covariates of a GLM with global AR(1) auto correlation correction and high-pass filter of 128 s. The GLM was comprised by nine regressors representing the experimental conditions (3 Reward conditions [Self, Fans and Non-fans], 3 Effort-only conditions after each rewarded ones and 3 Cue-only conditions) during the entire trial (6.5 s) and two regressors representing the movie clips of the participants’ own fan group and a neutral fan group with the exact stimulus duration (Mean: 7.06 s SD: ± 1.53 s). Furthermore, the six movement parameters obtained from realignment and nine regressors describing the respiratory cycle using RETROICOR^95^ were included as regressors of no interest to account for residual movement variance and respiration related effects (PhysIO Toolbox^96^). At the first level t-contrasts of each Reward vs. Effort-only conditions were created. These contrasts were used at the second level to assess specific brain activity related to each Reward condition, controlling for the effort. Therefore, the Effort-only condition was used as a high-level baseline contrast at the first level, so that we could control for the motor aspect of the task and focus on the motivational aspect at the second level.

At the second-level, one sample t-tests of each Reward vs. Effort-only condition (i.e. [Fans – Effort-only], [Self – Effort-only] and [Non-fans – Effort-only]) were performed for an exploratory evaluation of different brain areas related to each Reward condition. These whole-brain maps were inspected with a cluster-defining threshold of *p* < 0.001, to avoid higher number of false positives^97^ with cluster wise FWE correction as implemented in SPM 12 based on Random Field Theory^98^. Next, common brain areas related to monetary reward to self, ingroups and non-ingroups were evaluated controlling for the effort. Because conventional conjunction analysis for between subjects effects cannot be performed with factorial designs in SPM, binarized t-contrasts maps (voxel level *p* < 0.001, cluster FWE corrected *p* < 0.05) of each Reward vs. Effort-only conditions were subjected to a conjunction analysis, by employing the logical AND operator between the cluster level thresholded contrasts ([Self – Effort-only] AND [Fans – Effort-only] AND [Non-fans – Effort-only]. This approach revealed voxels that were consistently and significantly activated in all three Reward versus Effort-only contrasts (i.e., significant in the global conjunction of these contrasts^57^).

Specific brain areas related to each Reward condition were assessed at the second-level with paired t-tests of the first-level contrast of Reward vs. Effort-only conditions (e.g. [Fans – Effort-only] – [Non-fans – Effort-only]). As before, the whole-brain maps were inspected with a threshold of *p* < 0.001 with cluster wise FWE correction as implemented in SPM 12^98^. Considering our *a priori* hypotheses, the same procedure of previous studies^14,35,37^ were used, and SVC were applied in *a priori* ROIs at whole-brain voxel threshold of *p* < 0.005, uncorrected.

Furthermore, functional connectivity (FC) was evaluated through seed to voxels BSC FC analysis with BASCO toolbox^99^. BSC FC was originally proposed by Rissman *et al*.^100^ and recently have been suggested more appropriate for event-related designs than psychophysiological interaction methods^58^. The mOFC cluster revealed by the conjunction analysis between each Reward > Effort-only contrast was used as seed-ROI (*see Results*). *z*-transformed correlation maps for each Reward condition were used in a second level paired *t*-tests. These maps were inspected with a threshold of *p* < 0.005 (uncorrected) and SVC was applied in *a priori* ROIs.

*A priori* ROIs included SCC, VS and mOFC. The SCC ROI MNI coordinate (0, 26, −5) was determined by previous studies^14,101^. As for the mOFC and VS, ROIs centres MNI coordinates were obtained from meta-analyses, being (−2, 40, −4) and (12/−12, 10, −6), respectively^20,54^. A 10 mm sphere centred on the above coordinates was used as the search volume for every ROIs. All reported coordinates are in Montreal Neurological Institute space. Labelling of brain regions was based on the Harvard-Oxford probabilistic atlas and standard anatomical criteria^102^. The datasets generated in the current study are available from the corresponding author on reasonable request.

## Electronic supplementary material

Supplementary information
