# Supplementary material for: Neural bases of ingroup altruistic motivation in soccer fans
Source: Sci Rep. 2017 Nov 23;7:16122. doi: 10.1038/s41598-017-15385-7 (PMC5700961; doi:10.1038/s41598-017-15385-7)
Supplement: Supplementary file 1 — Supplementary information [file 41598_2017_15385_MOESM1_ESM.pdf]

Title:

Neural bases of ingroup altruistic motivation in soccer fans

Authors: Tiago Bortolini[a,b], Patrícia Bado[a,b], Sebastian Hoefle[a], Annerose Engel[a,c,d], Roland Zahn[e] , Ricardo de Oliveira Souza[a] and Jean-Claude Dreher[f,1] and Jorge Moll[a,1]

Author affiliations:
[a] Cognitive and Behavioral Neuroscience Unit, D'Or Institute for Research and Education, Rio de Janeiro
[b] Graduate Program in Morphological Sciences, Federal University of Rio de Janeiro
[c] Clinic for Cognitive Neurology, University of Leipzig, Germany,
[d] Max Planck Institute for Human Cognitive and Brain Sciences, Leipzig, Germany
[e] Institute of Psychiatry, Psychology & Neuroscience, Department of Psychological Medicine, Centre for Affective Disorders, King's College London, London, SE5 8AZ, UK
[f] Neuroeconomics, Reward and Decision-making Team, Institut des Sciences Cognitives Marc Jeannerod, Centre National de la Recherche Scientifique, 69675 Bron, France
[1]Authors declare equal senior contribution

Corresponding author:
Jorge Moll
D'Or Institute for Research and Education (IDOR)
Rua Diniz Cordeiro, 30
22281-100
Rio de Janeiro, Brazil
Tel +55 21 3883-6000
e-mail: jorge.moll@idor.com

Keywords:
Ingroup cooperation; altruism; fMRI; soccer fandom

SUPPLEMENTARY MATERIAL

Supplementary Methods

Detailed description of psychometric measures

Group identification has long been studied as one of the chief constructs of Social Identity Theory (1, 2), therefore having several available measures, differently from each other (3–5). We choose a validated measure of identification with soccer fans (6), the Football Supporter Team Identification Scale, which is composed of seven items that must be answered on a 10-point Likert scale in which 1 = *strongly disagree* and 10 = *strongly agree*.

Psychological kinship measures the extent to which an individual perceives other group members as family. It has three items and has been previously correlated to identity fusion and extreme pro-group behaviors (7). This construct has a direct relationship with evolutionary theories, being hypothesized as one of the proximal mechanisms to explain in-group cooperation with non-relatives (8).

The construct of entitativity (9), refers to the perception of a collection of persons as being bonded in a coherent unit. We used the measure created by Rüsch (2009), inspired on the study by Lickel and collaborators (2001) and based on Campbell (1958) and Rothbart and Park (2004) suggested components of entitativity: similarity, common fate and goals, and being a distinguishable and recognizable group in society. The measure has four items, one related to the perceived entitativity of a focal group and the other three related to each of the aforementioned components.

Detailed depiction of the fMRI task

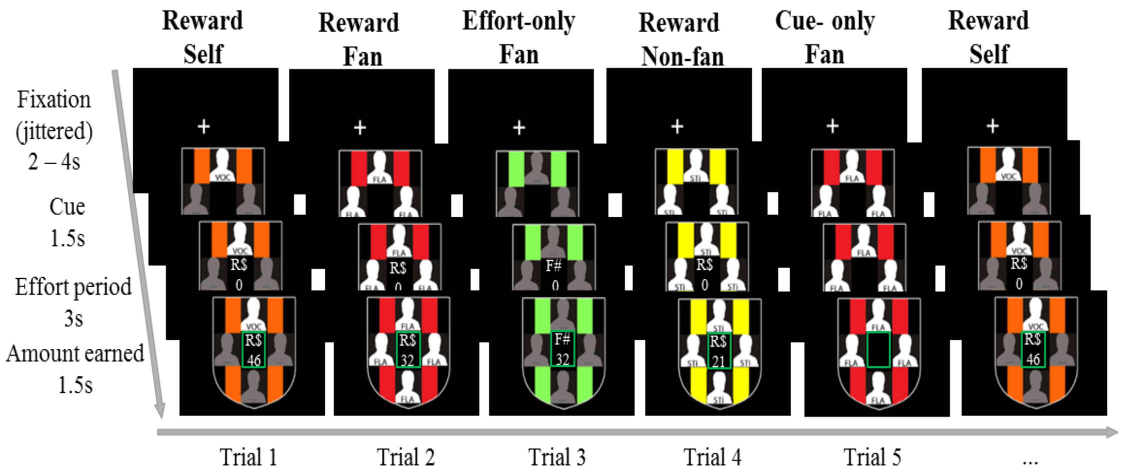

Figure S1. fMRI task design with an example of each condition. A cue indicating trial type was presented after a fixation cross jitter period. After cue presentation, participants pressed the handgrip dynamometer (effort period for Reward and Effort-only conditions) or passively observed the cue in Cue-only conditions. Real-time feedback of the amount earned during the effort period was provided, followed by a green outline indicating the end of the trial and the amount earned. Each condition was pseudo-randomly presented and the Effort-only condition was presented after 1/3 of each Reward condition. The cue for the Effort-only condition was always the same, indicating that participants should employ the same effort (as indicated by the number at the centre) as in the preceding Reward trial. The letters on the silhouettes stands for: VOC, "*você*" (Self); FLA, "*Flamengo*" (one of the soccer teams); STI, "*Sem Time*" (Non-fans).

Supplementary Results

Effects of self-concerned and altruistic reward conditions versus Effort-only

One-sample *t*-tests were performed to compare BOLD responses for each condition comparing Reward vs. Effort-only tasks. This high-level contrast enabled a tight control for effects of physical effort, while retaining the specificity of the motivation to obtain rewards for self and others. Whole-brain cluster FWE correction of each contrast showed robust BOLD responses in *a priori* ROIs as well in additional regions (Figures S1-S3 and Tables S1-S3). However, the interaction contrasts only indicated significant effects in the mOFC and the VS for (Self – Effort-only) > (Non-fan – Effort-only) (see Table 1 in the main text).

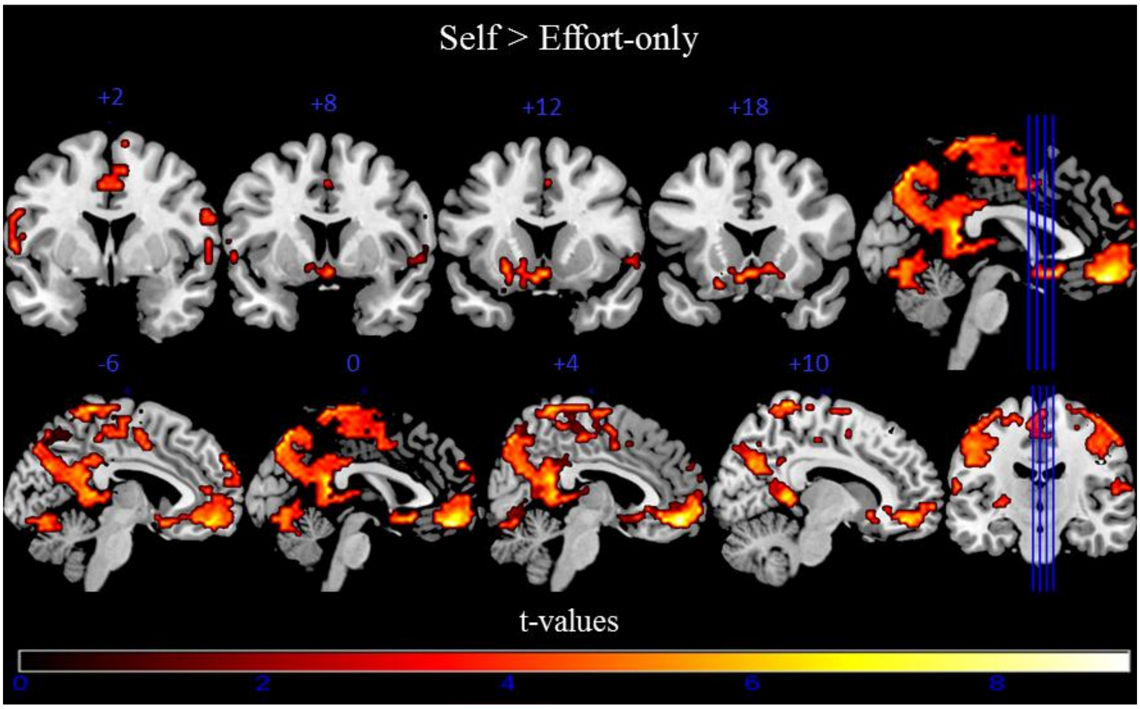

Figure S2. *t*-contrast maps of Reward self > effort only. All maps displayed at $p > .001$, cluster corrected FWE, k = 46.

Table S1. Whole-brain activations for Self > Effort-only contrast at *p* > .001, cluster corrected FWE (k = 46).

SELF > EFFORT-ONLY

**Region Name**	**Extent**	**Cluster peak t-value**	**MNI Coordinates x**	**y**	**z**
Frontal cortex (medial)	592	8.57	3	50	-13
Subcallosal cortex	592	5.69	0	20	-10
Cingulate gyrus (anterior division)	592	5.30	-12	44	8
	979	6.89	0	-43	17
Precuneus	979	6.27	0	-73	50
	979	6.22	12	-64	29
Precentral gyrus	2129	6.16	-54	-10	38
	2129	6.11	27	-25	53
Postcentral gyrus	2129	5.92	-24	-31	71
Lingual gyrus	186	5.67	-3	-76	-13
Occipital pole	186	4.04	-18	-91	-13
Planum temporale	94	5.59	63	-1	5
Opercular cortex (central)	94	4.51	60	-19	14
Frontal pole	78	5.28	-3	59	29
Insular cortex	73	3.93	-39	-4	-1

Note. Table lists all local maxima separated by more than 20 mm. Anatomical labelling according to the Harvard-Oxford Atlas as implemented in bsmp view toolbox.

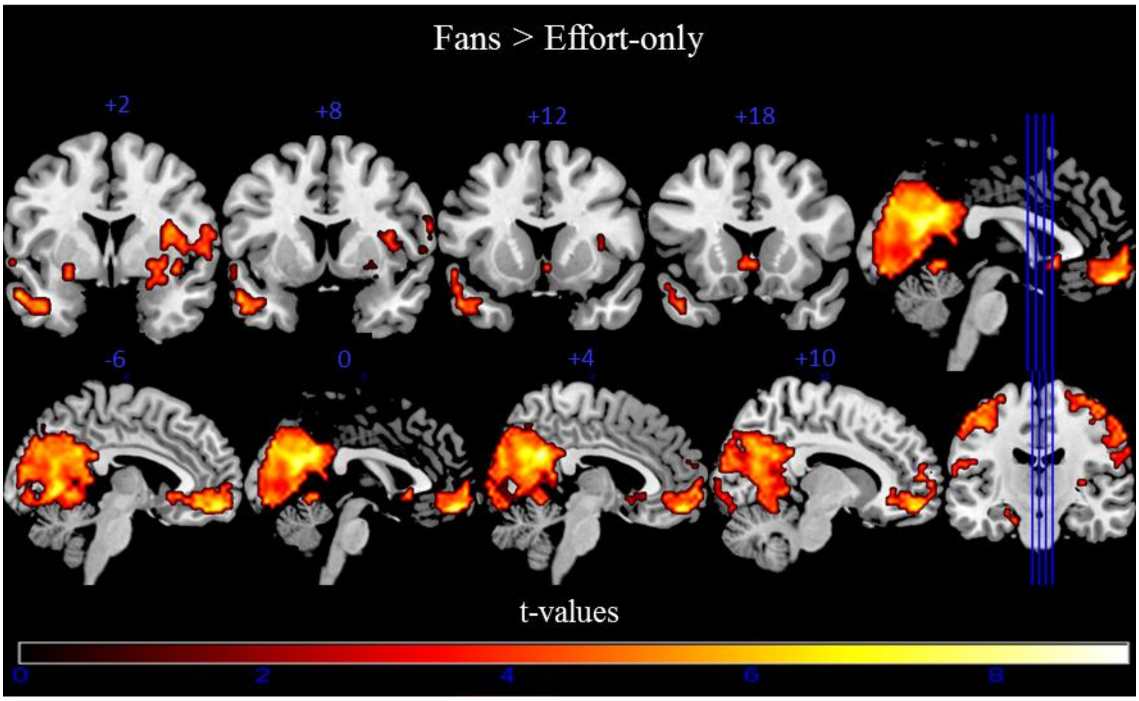

Figure S3. *t*-contrast maps of Reward Fan > effort only. All maps displayed at $p > .001$, cluster corrected FWE, k = 39

Table S2. Whole-brain activations for Fans > Effort-only contrast at $p > .001$, cluster corrected FWE (k = 39).

FANS > EFFORT-ONLY

Region Name	Extent	Cluster peak *t*-value	MNI Coordinates x	y	z
Cingulate gyrus (posterior division)	2586	8.42	3	-52	26
Lingual gyrus	2586	7.91	-9	-58	-4
Precuneus	2586	6.44	9	-70	35
Frontal cortex (medial)	521	8.01	-3	53	-13
Postcentral gyrus	681	7.70	42	-28	47
Precentral gyrus	695	7.03	-33	-25	56
	681	7.10	30	-22	68
	336	4.21	60	2	14
Supramarginal gyrus (anterior division)	681	6.08	57	-22	26
Insular cortex	336	7.14	36	-1	11
	336	4.81	39	-1	-10
Postcentral gyrus	695	6.72	-54	-22	47
Parietal operculum	695	5.20	-54	-25	20
Middle temporal gyrus (anterior division)	210	6.45	-54	-7	-19
Temporal pole	210	4.53	-42	14	-31
	210	3.93	-54	14	-13
Occipital cortex (superolateral division)	43	4.83	45	-61	29
Amygdala (left)	76	4.70	-27	-1	-13

Note. Table lists all local maxima separated by more than 20 mm. Anatomical labelling according to the Harvard-Oxford Atlas as implemented in bsmp view toolbox.

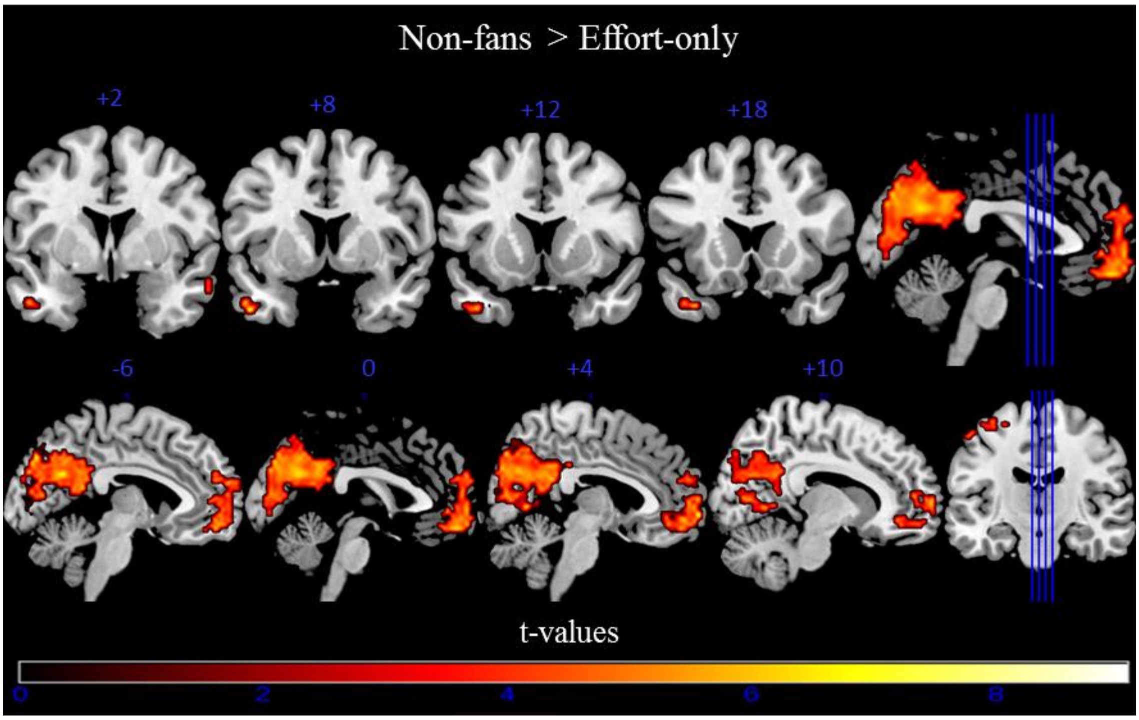

Figure S4. *t*-contrast maps of Reward Non-fan > effort only. All maps displayed at p > .001, cluster corrected FWE, k = 36.

Table S3. Whole-brain activations for Non-fans > Effort-only contrast at p > .001, cluster corrected FWE (k = 36).

NON-FANS > EFFORT-ONLY

**Region Name**	**Extent**	**Cluster peak *t*-value**	**MNI Coordinates x**	**y**	**z**
Precuneus	1054	6.75	-3	-64	26
Cuneus	1054	5.99	-3	-82	35
Lingual gyrus	1054	5.73	6	-64	2
Middle temporal gyrus (anterior division)	37	6.62	60	-7	-22
Frontal cortex (medial)	417	5.95	-3	53	-13
Frontal pole	417	5.38	9	65	5
Postcentral gyrus	132	5.37	-48	-25	59
	132	3.60	-30	-28	71
	64	5.06	-24	-31	56
Temporal pole	44	5.11	-36	17	-34
Occipital cortex (superolateral division)	49	4.91	-42	-64	26

Note. Table lists all local maxima separated by more than 20 mm. Anatomical labelling according to the Harvard-Oxford Atlas as implemented in bsmp view toolbox.

Beta parameters for the left VS *a priori* ROI for each Reward vs. Effort-only contrasts

To further explore the null result in the VS for the interaction contrast [Fans – Effort-only] > [Non-fans – Effort-only], we extracted the parameter estimates from the *a priori* left VS ROI (-12, 10, −6)[13] for all the three Reward vs. Effort-only contrasts (Fig. S5). Although the mean beta values were in line with our hypotheses, the effect in the VS for the interaction contrast Fans > Non-Fans did not reach significance due to the high standard errors.

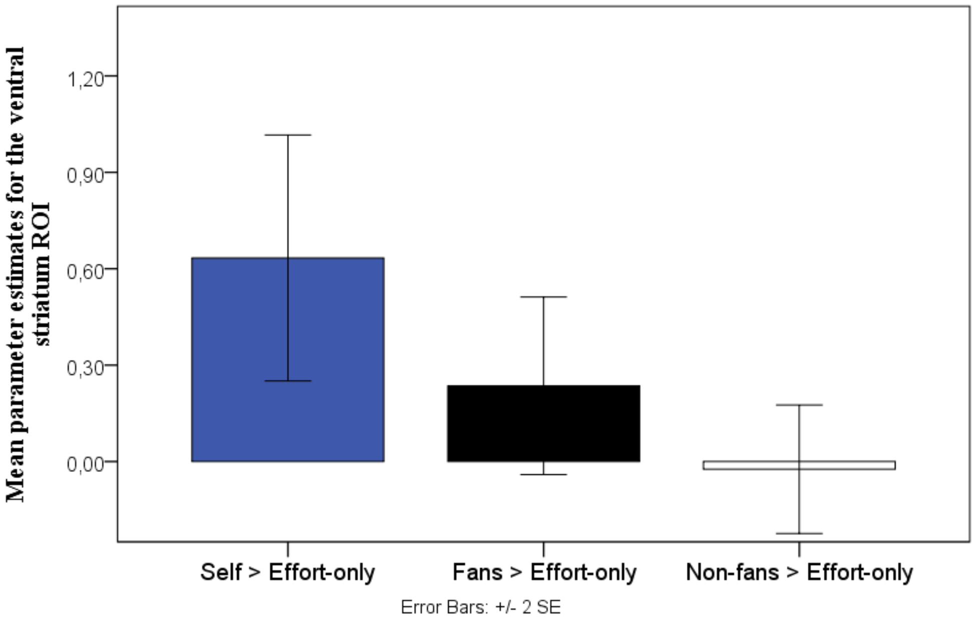

Figure S5. Parameter estimates for the left ventral striatum *a priori* ROI for each Reward vs. Effort-only contrast.

Differential effects for Self and Altruistic Reward conditions: Effects in the left mid-insular cortex.

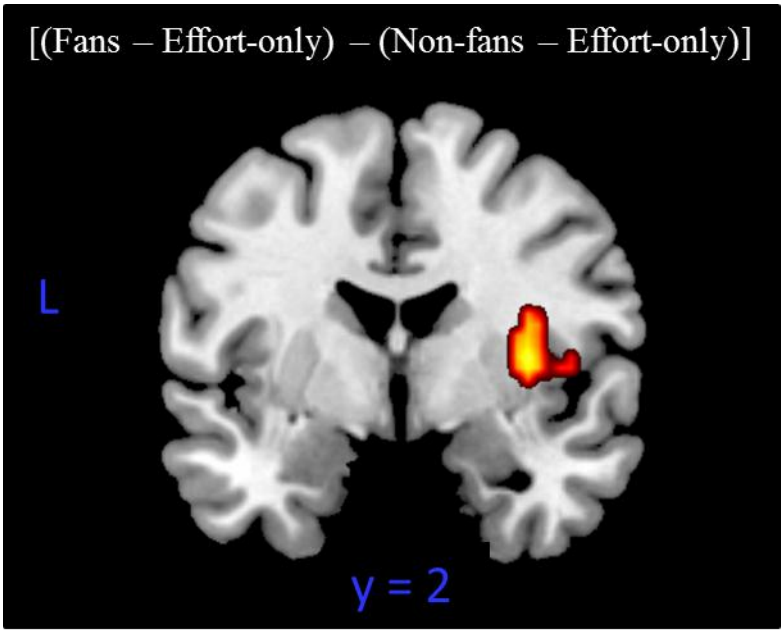

Figure S6. Whole-brain FWE cluster correction for multiple comparisons ($p < .001$; k = 37) of [(Fans - Effort-only) > (Non-fans - Effort-only)] contrast

References

1. Tajfel H, Turner JC (1979) An integrative theory of intergroup conflict In Psychology of Intergroup Relations. *The Social Psychology of Intergroup Relations.*, eds Austin WG, Worchel S (Brooks-Cole, Monterey, CA), pp 33–47.

2. Ellemers N, Spears R, Doosje B (2002) Self and Social Identity. *Annu Rev Psychol* 53(1):161–186.

3. Postmes T, Haslam SA, Jans L (2013) A single-item measure of social identification: Reliability, validity, and utility. *Br J Soc Psychol* 52(4):597–617.

4. Leach CW, et al. (2008) Group-level self-definition and self-investment: A hierarchical (multicomponent) model of in-group identification. *J Pers Soc Psychol* 95(1):144–165.

5. Cameron JE (2004) A Three-Factor Model of Social Identity. *Self Identity* 3(3):239–262.

6. Wachelke JFR, de Andrade AL, Tavares L, Neves JRLL (2008) Mensuração da identificação com times de futebol: evidências de validade fatorial e consistência interna de duas escalas. *Arq Bras Psicol* 60(1):98–110.

7. Buhrmester MD, et al. (2012) My Group's Fate Is My Fate: Identity-Fused Americans and Spaniards Link Personal Life Quality to Outcome of 2008 Elections. *Basic Appl Soc Psych* 34(6):527–533.

8. Crespi B, Summers K (2014) Inclusive fitness theory for the evolution of religion. *Anim Behav* 92:313–323.

9. Campbell DT (1958) Common fate, similarity, and other indices of the status of aggregates of persons as social entities. *Behav Sci* 3(1):14–25.

10. Rüsch N, et al. (2009) Ingroup perception and responses to stigma among persons with mental illness. *Acta Psychiatr Scand* 120(4):320–8.

11. Lickel B, et al. (2000) Varieties of groups and the perception of group entitativity. *J Pers Soc Psychol* 78(2):223–246.

12. Rothbart M, Park B (2004) The Mental Representation of Social Categories: Category Boundaries, Entitativity, and Stereotype Change. *The Psychology of Group Perception: Perceived Variability, Entitativity, and Essentialism*, eds Yzerbyt V, Judd CM, Corneille O (Psychology Press, New York, NY), pp 79–100.

13. Liu, X., Hairston, J., Schrier, M. & Fan, J. Common and distinct networks underlying reward valence and processing stages: A meta-analysis of functional neuroimaging studies. Neurosci. Biobehav. Rev. 35, 1219–1236 (2011).
